# Mitochondrial transfer and dynamics configures neural physiology through intercellular communications

**DOI:** 10.1002/alz70855_102364

**Published:** 2025-12-23

**Authors:** Sara Yunta‐Sánchez, Regina Mengual‐Fenollar, Juan Pedro Bolaños, Rubén Quintana‐Cabrera

**Affiliations:** ^1^ University of Salamanca, Salamanca, Salamanca, Spain; ^2^ Cajal Institute, Madrid, Madrid, Spain; ^3^ Cajal Institute, Madrid, Spain

## Abstract

**Background:**

In the nervous system, mitochondria can be transferred between neural cells through intercellular tunneling nanotubes (TNTs), microvesicles, or as free organelles. This transfer not only alters the mitochondrial content and respiration of recipient neural cells but also triggers a profound rewiring of their physiology, with glial cells and immune responses playing key roles in this reconfiguration.

**Method:**

Primary co‐cultures of neurons and glial cells, along with in vivo analysis of mitochondrial transfer in mouse brains, were monitored using kinetic microscopy, flow cytometry, and metabolic flux analyses to explore the physiological changes in neural cells. Mitochondrial DNA (mtDNA) transmission was tracked through RT‐PCR and ARMS‐PCR to examine hierarchical transfer and acquisition.

**Result:**

Communication between neural cells, particularly through TNTs, shows dynamic mitochondrial transfer, regulated by mitochondrial transport, fusion, and fission events. These events respond to structural and signaling changes in intercellular communication, mainly via TNTs. As a result, transmitted mitochondria reconfigure the content, metabolism, and mtDNA composition in recipient neurons and astrocytes. Notably, we observe a significant role of microglia and astrocytes upon mitochondrial acquisition in mouse brains, suggesting inflammatory events that may coordinate mitochondrial transfer as key regulators of metabolic rewiring and cognitive effects in the nervous system.

**Conclusion:**

Our findings provide evidence that a multilayered mitochondrial transfer is a critical mechanism for reconfiguring neural metabolism, immune responses, and overall neural physiology.

